# Pediatric Emergency Agitation Care Enhancement: Protocol for a Prospective Mixed Methods Study

**DOI:** 10.2196/92452

**Published:** 2026-04-21

**Authors:** Lillian Klein, Wendy Pomerantz, Teresa Pestian, Yin Zhang, Drew Barzman, Lisa Vaughn, Nancy Daraiseh, Ryan Siders, Holly Hanson, Bijan Ketabchi, Lynn Babcock

**Affiliations:** 1 Emergency Medicine, Cincinnati Children's Hospital Medical Center, 3333 Burnet Ave, Cincinnati, OH, United States, 1 513-636-7966; 2 Children's Hospital of Philadelphia, Philadelphia, PA, United States

**Keywords:** emergency service, hospital, pediatrics, mental disorders, behavioral symptoms, agitation, risk assessment, aggression, restraint, physical, qualitative research, mixed methods

## Abstract

**Background:**

Children and adolescents presenting to emergency departments (EDs) with mental and behavioral health (MBH) concerns frequently exhibit agitation, which poses safety risks for patients and staff and strains ED resources. Validated tools for agitation risk stratification in pediatric MBH populations are lacking, and evidence-based, risk-informed management strategies remain underdeveloped. Addressing these gaps is critical to reducing the need for emergent interventions, enhancing safety, and optimizing care delivery.

**Objective:**

This study aims to (1) evaluate the predictive ability of a brief pediatric agitation risk prediction tool, a 5-item version of the Brief Rating of Aggression by Children and Adolescents (BRACHA-S), and (2) codevelop an associated risk-based management plan bundle.

**Methods:**

This is a single-center prospective mixed methods study. First, we conducted a prospective cohort study enrolling children and adolescents aged 5 to 18 years who presented to the ED with MBH concerns. At ED triage, nurses completed the BRACHA-S risk assessment tool. The primary outcome, agitation requiring intervention, was defined as the use of pharmacologic agents, physical restraints, or mechanical restraints for events of agitation or aggression, as extracted from the electronic health record and confirmed through chart review. Second, we will use qualitative participatory methodologies, including key informant interviews, group-level assessments, and participatory design workshops, to codevelop a multifaceted, tiered, risk-based management plan designed to mitigate agitation and aggression in pediatric ED patients. Insights will be gathered from diverse stakeholders, including patients, parents or guardians, and members of the care team.

**Results:**

Funding was secured in July 2024. Initial data collection commenced in October 2024 and is projected to conclude in 2026. For aim 1, enrollment was completed on November 1, 2025, achieving the targeted sample size of 472 participants. We hypothesize that the BRACHA-S will demonstrate strong predictive validity (area under the curve >0.70) for agitation requiring intervention. Data collection for aim 2 began in October 2025, with analysis and results anticipated by June 2026. As of April 2026, 10 patients and parents/guardians were enrolled for key informant interviews, 30 staff members were enrolled for group-level assessment sessions, and 9 participants were enrolled for participatory design workshops. For this aim, we will codevelop a stakeholder-informed, tiered, risk-based agitation management pathway aligned with BRACHA-S risk strata.

**Conclusions:**

This study outlines a pragmatic approach to early agitation risk identification and the management of agitation in pediatric ED settings. If BRACHA-S demonstrates predictive validity and is paired with a stakeholder-informed, tiered management pathway, this strategy could fill critical gaps in validated tools and structured workflows, ultimately enabling timely, least-restrictive interventions and improving patient and staff safety.

## Introduction

The emergency department (ED) plays a pivotal role in screening children with acute mental and behavioral health (MBH) concerns. In the United States, over the past decade, ED encounters for pediatric MBH concerns have increased by 60%, reaching 13% of total visits in 2020 [1-3]. In a subset of these encounters, up to 10% involve agitation or aggression requiring intervention (ARI), inclusive of the use of pharmacologic, physical, or mechanical restraints [4-8]. Agitation poses safety risks to patients and staff and strains ED resources because agitated patients often require staff for 1-on-1 observation and designated psychiatric-safe rooms. Predicting patient agitation is essential to optimize care; however, validated agitation risk-stratification tools for children and adolescents cared for in the ED are lacking [9].

The Brief Rating of Aggression by Children and Adolescents (BRACHA) is a 14-item assessment conducted during ED mental health evaluations and is highly predictive of aggressive behaviors during inpatient psychiatric admission [10,11]. Typically completed by trained MBH specialists 1 to 2 hours after arrival at the ED or before admission, the BRACHA’s delayed administration, lengthy nature, and requirement for specialist completion render it ineffective as an early triage tool for predicting aggression in the ED. In a 9-year retrospective cohort study involving 32,091 children and adolescents presenting to the ED with MBH concerns, a shortened 5-item version of BRACHA (BRACHA-S; Table 1) demonstrated strong discriminative power (area under the curve 0.80) for predicting ARI in the ED [8]. This preliminary work suggests that a brief risk prediction tool may support earlier identification of children at risk for agitation and aggression.

**Table 1.** Items of the 5-item Brief Rating of Aggression by Children and Adolescents and scoring scheme.

Item description	Score (no=0 or yes=1)
History of psychiatric hospitalizations	No or yes
Frequency of physically aggressive acts toward others	Never or often
Threats or physical aggression toward self or others in the past 24 hours	No or yes
Impulsivity or agitation during evaluation	No or yes
Intrusiveness toward others during evaluation	No or yes

In addition to the BRACHA, other brief observational tools have been proposed to identify imminent aggression in various care settings [12]. The Brøset Violence Checklist (BVC), created for psychiatric inpatient units, predicts violent incidents within 24 hours by assessing 6 behaviors associated with escalating agitation (confusion, irritability, boisterousness, verbal threats, physical threats, and attacking objects) [13-16]. The BVC has been used in more than 62 studies across various settings, primarily in adult and inpatient psychiatric populations [17]. Another tool, the STAMP framework, developed from qualitative work with emergency nurses, focuses on 5 observable behaviors that may signal escalating risk: staring, changes in tone or loudness of voice, visible anxiety, mumbling, and pacing [18]. To date, STAMP has not been systematically evaluated in ED patient populations, and its predictive properties are unknown. Conceptually, both STAMP and the BVC can be applied at triage and repeated throughout an ED visit as brief, momentary assessments of agitation and violence risk. However, neither tool has been prospectively validated in pediatric ED settings for predicting the need for agitation-related interventions, underscoring the need for rigorously tested, evidence-based violence and agitation risk assessment tools for pediatric emergency care.

Current guidelines for pediatric agitation management emphasize early, least-restrictive care, including verbal de-escalation and environmental modification, to reduce the need for emergent pharmacologic or physical interventions while maintaining safety [9,17,19-23]. As recommended by the Joint Commission, patients in the ED with MBH concerns are routinely screened for suicide risk using brief, validated tools at triage [24-27] to stratify the risk of self-harm and initiate tiered responses that enhance safety. However, these approaches fail to identify individuals who may pose a risk of aggression toward others or property [28]. Collectively, these findings highlight the need for a stakeholder-informed, risk-based approach to agitation and aggression, guided by a validated risk assessment at triage and linked to a tiered ED management plan, which could support safer and more consistent care while streamlining downstream decision-making and resource use.

The Pediatric Emergency Agitation Care Enhancement study is a single-center, prospective, mixed methods study designed to address this gap. The objectives are to evaluate a brief pediatric agitation risk prediction tool (BRACHA-S) and to develop an associated risk-based management plan bundle. The primary quantitative outcome is the identification of an ARI event. Qualitative participatory methods will be used to co-design a tiered, risk-based care plan. We will triangulate prospectively collected quantitative and qualitative data to fulfill our objectives through the following specific aims:

1. Aim 1 (quantitative) is to evaluate the predictive ability of the BRACHA-S administered by non-MBH specialists early in the ED course for assessing agitation or aggression in children with MBH concerns by conducting a prospective cohort study. This study hypothesizes that the BRACHA-S will have robust predictive ability (area under the curve >0.70) for ARI.
2. Aim 2 (qualitative) is to develop a multifaceted, tiered, risk-based management plan designed to mitigate agitation or aggression by gathering insights from a diverse group of stakeholders, including patients, parents or guardians, and members of the care team, using qualitative methodologies, including group-level assessment (GLA) sessions, key informant (KI) interviews, and participatory design workshops (PDWs). The product to be created in this study is an ED risk-based agitation or aggression management plan.

## Methods

### Overview

This is a single-center, prospective, mixed methods study, funded by the Place Outcomes Research Award grant. The study period spans July 2024 to July 2026. Reporting of the quantitative cohort for aim 1 will follow the Strengthening the Reporting of Observational Studies in Epidemiology (STROBE) guidelines and incorporate relevant items from the Transparent Reporting of a Multivariable Prediction Model for Individual Prognosis or Diagnosis (TRIPOD) statement [29,30]. Reporting of the qualitative participatory aim 2 will follow the Standards for Reporting Qualitative Research (SRQR) [31].

### Aim 1: Quantitative Prospective Cohort

#### Approach and Population

This was a prospective cohort study that enrolled children and adolescents presenting to the ED with MBH concerns, as identified by the triage nurse and documented with a chief complaint of “psychiatric evaluation,” “behavior concern,” “aggression,” or a similar MBH-related complaint, in accordance with predefined inclusion and exclusion criteria in Textbox 1.

Textbox 1. **Aim 1 inclusion and exclusion criteria.**

**Inclusion criteria**

- Patients aged 5-18 years
- Presentation with a mental and behavioral health concern

**Exclusion criteria**

- Life-threatening emergencies
- Need for immediate intervention to mitigate agitation or aggression upon arrival at the emergency department

#### Study Procedures

Before recruitment, nursing staff underwent training on the BRACHA-S, BVC, and STAMP tools, including recommended assessment methods for each tool, with regular retraining throughout the recruitment period. Screening windows for the 1-year recruitment period were generated using a random block method based on historical MBH patient arrival data and Clinical Research Coordinator (CRC) coverage. During these windows, CRCs reviewed the ED track board and triage documentation to identify eligible patients and approached a convenience sample. Triage or initial care team nurses completed the BRACHA-S and the initial BVC and/or STAMP shortly after patient arrival, facilitated by the CRC using tablets. Repeat BVC or STAMP scores were completed by care team members, including behavioral health specialists, patient care attendants, nurses, and clinicians, at regular intervals (approximately every 3 to 4 hours) during the patient’s ED stay. Declines to complete the tools were documented.

Additional data were extracted from the electronic health record (EHR), including demographics, restraint application, administered medications, MBH specialist-acquired BRACHA scores and diagnoses, length of stay, disposition, ED diagnosis, and relevant timestamps, and merged into a comprehensive database. Staff injury data were collected using a tablet-based questionnaire adapted from a prior injury monitoring tool [32]. CRCs asked staff involved in the patient’s care to complete the survey approximately once per shift during the patient’s ED stay. In addition, injury events linked to enrolled patients and reported to our institutional injury reporting system were obtained from the institution’s occupational safety team and uploaded into the study database.

#### Study Variables and Outcomes

The primary predictor was the BRACHA-S total score, with additional predictors including the initial BVC and STAMP scores recorded at triage. The primary outcome, ARI, was defined as agitation or aggression posing a risk of harm to self or others that resulted in pharmacologic management or physical or mechanical restraint during the ED visit. Intervention data were extracted from the EHR, including medications administered, use of the agitation order set, and restraint orders, and were confirmed through chart review for each agitation or aggression event. Exploratory outcomes to be assessed will include severe ARI (intramuscular pharmacologic interventions and physical or mechanical restraints), changes in BVC or STAMP scores over time, and staff injuries categorized by type, mechanism, and severity. Key covariates will include age, sex, race, ethnicity, insurance status, presenting complaint, prior MBH visits, ED length of stay, and disposition.

#### Statistical Analysis Plan

##### Aim 1 Sample Size

On the basis of prior analysis, we observed rising proportions of ARI, with rates of 1.5%, 3%, 8%, 17%, 30%, and 45% corresponding to BRACHA-S scores of 0 to 5. The sample sizes across these BRACHA-S scores were proportioned at 0.8, 1, 1, 1, 1, 1, 1, 0.3, and 0.15, reflecting the expected relative distribution of patients across score categories. Sample size estimates were derived from logistic regression, aiming for a type 1 error rate of 5% and >80% power to detect pairwise differences across various score groupings. A sample size of 472 observations was estimated to detect differences across 4 score groups, with the estimated number of patients in each group as follows: scores 0 to 1 (199/472, 42.2% patients), score 2 (111/472, 23.5%), score 3 (111/472, 23.5%), and scores 4 to 5 (51/472, 10.8%). With more than 3500 MBH patients annually having documented BRACHA scores, a 12‐ to 18-month recruitment period using screening windows was expected to suffice.

##### Aim 1 Analysis

Patient characteristics, predictors, and outcomes will be summarized using descriptive statistics. Associations between demographic variables (eg, age category and sex) and ARI (primary outcome) will be examined using chi-square tests. The association between BRACHA-S scores and first ARI in the ED will be evaluated using logistic regression. Discriminative performance will be assessed with receiver operating characteristic curves and performance metrics (sensitivity, specificity, positive predictive value, and negative predictive value) at selected BRACHA-S thresholds. Calibration will be examined by comparing observed and predicted ARI rates across BRACHA-S risk groups. Sensitivity and subgroup analyses will be conducted to evaluate the robustness of the model when there is potentially population heterogeneity across age groups, sex, ED site, and workflow period. Supplementary models may include demographic variables and the initial BVC or STAMP scores as additional predictors.

Comparative analyses will explore how BRACHA-S, BVC, and STAMP scores relate to one another and differ in their ability to predict ARI. To assess the relationship between BRACHA-S and agitation-related behaviors over time, distributions and correlations between BRACHA-S and BVC or STAMP scores will be examined. A generalized linear mixed model with an appropriate count distribution (eg, Poisson distribution) and correlation structure will be considered to analyze associations between BRACHA-S and repeated BVC or STAMP scores during the ED stay, with demographic variables included as fixed effects. Additional exploratory analyses may include associations between BRACHA-S scores and staff injuries and interrater reliability of BRACHA-S component scores between nurses and MBH specialists. Analyses may be repeated using the severe ARI outcome, defined by intramuscular pharmacologic medications typically used for agitation or aggression and physical or mechanical restraints.

The extent and pattern of missing data for BRACHA-S, BVC, STAMP, outcomes, and key covariates will be described. Primary analyses will use available case data; if substantial missingness is identified for BRACHA-S or ARI, sensitivity analyses using appropriate methods such as multiple imputation may be conducted.

### Aim 2: Qualitative

#### Approach and Population

Aim 2 uses a constructivist, stakeholder-engaged qualitative and participatory design that combines individual interviews, GLA sessions, and PDWs to codevelop a risk-stratified agitation management pathway.

Stakeholders will be recruited using purposive sampling, through in-person, email, text message, or other virtual means, to capture diverse perspectives across patients, caregivers, and ED care team members. Up to 70 stakeholders will be recruited according to the criteria in Textbox 2, including approximately 10 to 15 patients and parents or guardians for KI interviews, 15 to 20 stakeholders per GLA session (up to 3 sessions), and up to 10 stakeholders for a PDW.

Textbox 2. **Aim 2 inclusion and exclusion criteria.**

**Inclusion criteria**

- Patients aged 5-18 years who have experienced care for mental and behavioral health concerns in an emergency department setting, and/or their parents or guardians
- Stakeholders involved in emergency department mental and behavioral health care processes (ie, clinicians, nurses, social workers, counselors, behavioral health specialists, and protective services personnel)

**Exclusion criteria**

- Non–English-speaking participants
- Patient stakeholders with a history of severe neurodevelopmental issues or other conditions that prevent optimal participation in discussions
- Parents or guardians who do not reside with the patient participant at least 50% of the time
- Patients who are in the custody of a county child welfare agency

#### Study Procedures

##### Overview

An overview of the study procedures involved in aim 2 is presented in Table 2, with further details provided later.

**Table 2.** Qualitative methodologies and objectives.

Item number	Content	Type or sample	Objective
1	Symptoms, treatment, and experiences with restraint for agitation	Up to 10 KI^a^ interviews	Conduct interviews with patients and their parents to gather insights to inform the development of a tiered agitation management plan
2a	In-depth understanding of responses and ideas from KI interviews	3 GLA^b^ sessions (~30 stakeholders per session; total ~90 stakeholders)	Provide additional context and insights on KI interview findings and engage a wide range of stakeholders in generating, analyzing, and prioritizing ideas
2b	Initial draft of the care management plan	Action-planning session (7-step GLA process; 10-12 individuals)	Synthesize findings from aim 1, GLA sessions, and KI interviews
3	Development of a tiered agitation care algorithm and tailored care pathways	5 participatory design workshop sessions (5-7 individuals per session)	Create a consensus-based pediatric agitation risk prediction tool and associated care plan bundle

a KI: key informant.

b GLA: group-level assessment.

##### KI Interviews

These interviews will gather open‐ended insights from MBH patients and their parents or guardians about calm‐promoting strategies and experiences with agitation-related care. Patients will be recruited through the ED, and interviews will be conducted in person or through secure web conferencing. Interviews will be conducted by trained members of the research team using a semistructured guide with open-ended questions and prompts to allow participants to elaborate on their experiences [33]. Each KI interview is expected to take approximately 20 to 40 minutes to complete.

##### GLA Method

GLA is a qualitative, participatory method designed to engage large groups of diverse stakeholders who generate, interpret, synthesize, and evaluate data in participatory sessions [33-35]. Stakeholders, including health care providers and other staff involved in MBH care, will be recruited through the ED and personal invitations to participate in virtual GLA sessions. Up to 3 sessions will be arranged, each with up to 20 unique participants. Sessions are hosted on a secure video-based platform and recorded. All participants will complete a brief survey capturing demographics (eg, age group, sex, role, and years in role).

A trained qualitative methodologist will facilitate the 7-step GLA process [33-35]. Each session will begin with an introduction outlining its purpose, confidentiality, recording guidelines, and group rules (step 1: climate setting). Stakeholders will respond to open-ended prompts on Padlet (version 223.3.0; Wallwisher Inc; an online platform that mimics flip charts) regarding symptoms, treatments, and experiences, with a focus on developing a plan to promote the least restrictive means of restraint for agitation (step 2: generating). Participants will then examine the totality of responses to the prompts (step 3: appreciating), followed by individual reflections on others’ contributions (step 4: reflecting). From the large group of stakeholders, smaller groups of 4 to 6 individuals will distill and summarize data from assigned prompts into themes to report out (step 5: understanding). The facilitator will keep a running list of themes in the participants’ own words. Themes will then be prioritized by participants (step 6: selecting). The study team will synthesize the findings across the 3 GLA sessions. Select GLA participants will be invited to a separate action-planning session (step 7: GLA) to inform them of the design priorities for the risk prediction tool and care plan bundle. Attendees (n=10-12) of the action-planning session will synthesize the findings from aim 1 and the GLA sessions, aiming to develop an initial draft of a care management plan for future work. Each GLA session is expected to take approximately 90 minutes to complete.

##### PDW Sessions

A group of 5 to 7 key stakeholders (involved in prior GLA sessions) will participate in up to five 2-hour PDW sessions, either virtually or in person [36-38]. These workshops will focus on collaboratively integrating quantitative and qualitative data to create a consensus-based pediatric agitation risk prediction tool and associated care plan bundle, emphasizing risk mitigation and least-restrictive interventions. Facilitated by a trained methodologist, the PDWs will use structured consensus-building techniques, idea generation, and iterative discussion to guide bundle development [39,40]. Personas representing diverse patient profiles (informed by quantitative and qualitative data) will be collaboratively developed to enhance the understanding of patient experiences and inform the design of tailored care pathways [41-44]. Stakeholders will iteratively refine the tool and care plan bundle through role-play, experiential activities, and discussion.

##### Research Teams and Qualitative Data Management

The multidisciplinary research team (emergency medicine, psychiatry, nursing, human factors, and qualitative methods) will leverage their collective experience with ED MBH care. Brief team debriefings and analytic notes will be used to reflect on roles and assumptions. All KI interviews, GLA sessions, action-planning sessions, and PDWs will be audio- and/or video-recorded using secure platforms. Recordings will be transcribed by trained staff or a Health Insurance Portability and Accountability Act (HIPAA)–compliant transcription service and checked for accuracy. Transcripts, Padlet responses, session notes, and other qualitative materials will be deidentified before analysis and managed using qualitative data analysis software on secure institutional servers.

##### Reimbursement

Participants will be reimbursed for time, effort, and travel. Participation will not be contingent on accepting payment.

### Statistical Analysis Plan

#### Aim 2 Sample Size

Drawing from previous work led by the coinvestigator (LV), it is anticipated that 1 to 3 GLA sessions involving 15 to 20 participants each will provide ample data for thematic analysis and meet the study’s objectives. Likewise, a group comprising 5 to 7 stakeholders is typically conducive to achieving consensus and finalizing the risk-stratified care pathway.

#### Aim 2 Analysis

KI interviews will be analyzed using an established thematic analysis approach, which uses a 6-step inductive process of coding and distilling patterns in the data (themes) [45,46]. Two analysts will code an initial subset of transcripts, develop a shared codebook, and apply it to the remaining interviews. Data generated in GLA sessions will be incorporated iteratively, with participants collaboratively analyzing and prioritizing ideas during the sessions. In the PDW, qualitative data, including field notes, participant discussions, writings, and session materials, will also be analyzed using thematic analysis [45,46]. Themes from KI, GLA, and PDW activities will be compared and integrated to inform the development and refinement of the multifaceted, tiered, risk-based management plan. Trustworthiness will be supported through triangulation across methods and stakeholder groups and the maintenance of an audit trail of analytic decisions. The final bundle will be determined through group consensus-building to reflect the collective insights and perspectives of stakeholders.

### Ethical Considerations

This study was approved by Cincinnati Children’s Hospital Institutional Review Board (2024-0413) and conducted in compliance with institutional review board and HIPAA regulations [47]. This study involves minimal risk to patients, families, and participating clinicians.

For aim 1, enrollment was conducted under a waiver of informed consent and HIPAA authorization. Electronic medical records were used to screen for eligibility. Identifiable data (eg, medical record number, visit time, demographics, and clinical data), agitation risk assessments (BRACHA-S, BVC, or STAMP), and linked staff injuries were stored in a REDCap (Research Electronic Data Capture) research database. No study-specific procedures altered clinical management, and interactions related to data collection occurred only with the treating team. Staff injury surveys were voluntary, and responses were recorded without personal identifiers other than clinical role. No compensation was provided for participation in aim 1.

For aim 2, written or electronic informed consent and, when applicable, assent will be obtained from patient and parent or guardian participants before the start of KI interviews. Health care team members and parents or guardians involved in GLA and PDW activities will participate under a waiver of documented consent. They receive a study information sheet and provide verbal consent or assent before study procedures. At the start of each group session, facilitators will review ground rules emphasizing confidentiality, respectful discussion, and voluntary participation. Participants may decline to answer any question or withdraw at any time without impact on clinical care or employment. Each participant will receive up to US $40 per session.

All study data, including EHR extracts, survey responses, and staff injury reports, will be stored in accordance with HIPAA and institutional data security policies [48]. Data will be entered into secure, password-protected REDCap databases or other approved institutional systems with role-based access. Identifiers will be removed or replaced with unique study codes at the earliest possible time, and linkage files will be stored separately in restricted-access locations. Only authorized study personnel will have access to identifiable data, and all reports and publications will present aggregate, deidentified results.

## Results

Funding was secured in July 2024. Enrollment for aim 1 was completed on November 1, 2025, with 472 participants enrolled. Data cleaning and preliminary descriptive summaries are underway, and complete analysis will be conducted in early 2026. For aim 2, stakeholder recruitment and qualitative data collection began in October 2025 and are ongoing, with completion anticipated by May 2026. As of April 2026, 10 patients and parents/guardians were enrolled for KIs, 30 staff members were enrolled for GLAs, and 9 participants have been enrolled for PDWs. Analyses for aim 2 will be finalized by July 2026, and results will be disseminated in subsequent publications.

## Discussion

### Anticipated Findings

This study protocol outlines a mixed methods approach to enhance care for children and adolescents presenting to the ED with MBH concerns by prospectively evaluating the predictive performance of the BRACHA-S tool at triage and developing a tiered, risk-based management plan. With completion of this study, and assuming adequate predictive performance, the results are expected to support BRACHA-S as an early risk-stratification tool for agitation in the ED for patients with MBH concerns and to yield a stakeholder-derived, tiered management plan ready for testing in future implementation studies. This risk-stratified approach has the potential to reduce reliance on emergent pharmacologic and physical interventions, improve patient and staff safety, and enhance operational efficiency through early recognition of elevated risk, more strategic allocation of ED resources, and more targeted interventions.

Evidence supporting brief agitation or violence risk tools in ED settings remains limited. Literature syntheses of studies conducted in adult EDs identify more than 10 brief violence risk tools and conclude that, although these instruments appear feasible for short-term risk stratification, psychometric evidence is sparse, heterogeneous, and insufficient to support recommending any single tool [49-52]. In the pediatric ED setting, far fewer instruments have been studied [53]. Studies on the Pediatric Violence/Aggression Assessment Tool [54] and the Dynamic Appraisal of Situational Aggression, Youth Version [55] suggest that higher scores are associated with increased aggression and greater restraint use in the ED; however, external validation is limited, and outcome definitions (eg, aggression events, restraint use, and medication use) vary across studies. These gaps underscore the need for rigorous testing of agitation risk tools such as BRACHA-S within real-world pediatric ED workflows. This study will also generate exploratory data on the BVC and STAMP framework in pediatric emergency care. BVC is validated for short-term aggression prediction in psychiatric units and has been implemented in adult EDs as part of workplace violence initiatives; however, pediatric ED performance data remain sparse [16,56,57]. STAMP, developed from qualitative observation in adult EDs, is widely cited in violence-prevention resources but lacks formal validity and reliability testing and has not been evaluated as a predictive tool in pediatric ED populations. Collecting these measures alongside BRACHA-S and ARI will provide comparative estimates to inform future validation studies and potential refinement of ED-specific agitation risk tools for children and adolescents.

Staff injury related to patient aggression is a central occupational safety concern in pediatric ED agitation care. National survey data show that 96% of pediatric emergency physicians have experienced workplace violence, and 10% have sustained injuries requiring medical attention or time off, with approximately half of the incidents unreported [58]. In pediatric inpatient settings, a high-risk flag identified a small subgroup that accounted for a disproportionate share of staff injuries [59], supporting exploration of whether ED risk stratification aligns with occupational risk. Furthermore, quality improvement work in inpatient settings suggests that staff injuries may be reduced through system-level responses, such as behavioral response teams for children and adolescents at risk of aggression [60]. Accordingly, this protocol will explore linkages among agitation risk scores and staff injury surveillance in the ED to inform future prevention strategies.

Current best-practice recommendations for management of agitated children or adolescents emphasize early, least-restrictive strategies, including verbal de-escalation, environmental modification, and proactive engagement of behavioral health resources, with stepwise escalation to medication and restrictive interventions only when needed to maintain safety [19,61,62]. Standardized pathways and order sets may reduce variation by clarifying roles, aligning team communication, and supporting timely selection of nonpharmacologic and pharmacologic options [9]. However, the evidence base remains heterogeneous and largely single-site, and few approaches explicitly integrate prospective risk stratification at triage to tailor the intensity of response. Results from this study may address this gap by pairing early risk identification with standardized, risk-appropriate actions in a pediatric ED setting.

Prior qualitative and mixed methods studies of pediatric behavioral emergencies describe persistent barriers to delivering least-restrictive care in ED settings, including variable staff training and confidence with de-escalation, unclear roles and communication during escalation, environmental constraints, and staff safety concerns [17,20,63]. Most published pediatric ED agitation pathways have been developed by multidisciplinary clinical teams, with limited description of patient or caregiver co-design [9]. Parent-inclusive stakeholder processes have been used to define pediatric ED agitation quality measures, demonstrating the feasibility and value of incorporating patient and caregiver perspectives in this domain [64]. In this protocol, participatory design methods extend this literature by incorporating stakeholder perspectives on risk and selection of management strategies to build a tiered approach for caring for agitated children and adolescents in pediatric ED settings.

### Strengths and Limitations

The integration of both quantitative and qualitative methodologies will provide a comprehensive understanding of predictive accuracy and contextual implementation needs. A key strength of this study is its pragmatic design, which evaluates the BRACHA-S tool in real-world ED settings using frontline nursing staff. This enhances the generalizability and potential scalability of the findings. The prospective cohort design supports robust evaluation of the predictive validity of the tool, while the participatory design methods ensure that the resulting care plan bundle is informed by the lived experiences and insights of diverse stakeholders, including patients, families, and care providers.

This study has several limitations. It is conducted within a single pediatric health system, which may limit generalizability. Aim 1 uses a convenience sample during predefined screening windows, and nurse refusal to complete BRACHA-S, BVC, or STAMP may introduce selection bias. ARI is ascertained from electronic orders and documentation, so incomplete recording of behaviors, brief holds, or de-escalation efforts may lead to outcome misclassification. Additional de-escalation interventions were not systematically recorded, limiting understanding of how agitation risk scores operate within the broader spectrum of agitation management. Exploratory analyses of BVC and STAMP are not powered for precise estimates of predictive performance. For aim 2, qualitative findings rely on an English-speaking, self-selected sample from a single institution, which may limit transferability to other settings.

### Conclusions

Early identification and intervention are critical for managing agitation and aggression in pediatric MBH patients; however, practical tools and workflows in emergency settings remain limited. This study positions BRACHA-S as a brief, nurse-administered triage tool for early quantification of agitation risk among children and adolescents presenting to the ED with MBH concerns. Paired with a stakeholder-informed, tiered management pathway, it establishes a structured foundation for safer, more consistent, and patient-centered agitation care in pediatric emergency settings.

## Supplementary material

10.2196/92452 Peer Review Report 1 Peer review report by Place Outcomes Research Award Review Committee, Cincinnati Children’s Hospital Medical Center.

## Abbreviations

ARI: aggression requiring intervention
BRACHA: Brief Rating of Aggression by Children and Adolescents
BRACHA-S: shortened 5-item version of Brief Rating of Aggression by Children and Adolescents
BVC: Brøset Violence Checklist
CRC: Clinical Research Coordinator
ED: emergency department
EHR: electronic health record
GLA: group-level assessment
HIPAA: Health Insurance Portability and Accountability Act
KI: key informant
MBH: mental and behavioral health
PDW: participatory design workshop
REDCap: Research Electronic Data Capture
SRQR: Standards for Reporting Qualitative Research
STAMP: Systems Theoretic Accident Model and Processes
STROBE: Strengthening the Reporting of Observational Studies in Epidemiology
TRIPOD: Transparent Reporting of a Multivariable Prediction Model for Individual Prognosis or Diagnosis

## References

[1] Lo CB, Bridge JA, Shi J, Ludwig L, Stanley RM (2020). Children’s mental health emergency department visits: 2007-2016. Pediatrics.

[2] Bommersbach TJ, McKean AJ, Olfson M, Rhee TG (2023). National trends in mental health-related emergency department visits among youth, 2011-2020. JAMA.

[3] Hoffmann JA, Stack AM, Samnaliev M, Monuteaux MC, Lee LK (2019). Trends in visits and costs for mental health emergencies in a pediatric emergency department, 2010-2016. Acad Pediatr.

[4] Foster AA, Porter JJ, Monuteaux MC, Hoffmann JA, Hudgins JD (2021). Pharmacologic restraint use during mental health visits in pediatric emergency departments. J Pediatr.

[5] Nash KA, Tolliver DG, Taylor RA (2021). Racial and ethnic disparities in physical restraint use for pediatric patients in the emergency department. JAMA Pediatr.

[6] Hoffmann JA, Corboy JB, Liu L (2024). Use of electronic health record-based measures to assess quality of care for pediatric agitation. Hosp Pediatr.

[7] Tang AS, Shieh MS, Pekow PS, Prentiss KA, Lindenauer PK, Westafer LM (2023). Treatment of pediatric behavioral health patients with intravenous and intramuscular chemical restraints: results from a nationwide sample of emergency departments. Acad Emerg Med.

[8] Ketabchi B, Babcock L, Zhang Y, Barzman D, Pomerantz WJ, Cincinnati Children’s Hospital Medical Center Prehospital Care Committee (2025). Predicting agitation in the emergency department. Pediatrics.

[9] Hoffmann JA, Pergjika A, Liu L (2023). Standardizing and improving care for pediatric agitation management in the emergency department. Pediatrics.

[10] Barzman DH, Brackenbury L, Sonnier L (2011). Brief Rating of Aggression by Children and Adolescents (BRACHA): development of a tool for assessing risk of inpatients’ aggressive behavior. J Am Acad Psychiatry Law.

[11] Barzman D, Mossman D, Sonnier L, Sorter M (2012). Brief Rating of Aggression by Children and Adolescents (BRACHA): a reliability study. J Am Acad Psychiatry Law.

[12] Väätäinen L, Björkqvist M, Li Y, Pelto-Piri V, Ferreira A, Lantta T (2025). Instruments for short-term (24 h) violence risk assessment and strategies for managing violence risk among adolescents with risk for violent behaviour: a systematic review. Int J Ment Health Nurs.

[13] Almvik R, Woods P, Rasmussen K (2000). The Brøset Violence Checklist: sensitivity, specificity, and interrater reliability. J Interpers Violence.

[14] Woods P, Almvik R (2002). The Brøset Violence Checklist (BVC). Acta Psychiatr Scand Suppl.

[15] Ilarda E, McIlveen P, Tynan A, Senz A (2024). Emergency department staff experiences of the Bröset Violence Checklist. J Adv Nurs.

[16] Hvidhjelm J, Berring LL, Whittington R, Woods P, Bak J, Almvik R (2023). Short-term risk assessment in the long term: a scoping review and meta-analysis of the Brøset Violence Checklist. J Psychiatr Ment Health Nurs.

[17] Lelonek G, Crook D, Tully M, Trufelli K, Blitz L, Rogers SC (2018). Multidisciplinary approach to enhancing safety and care for pediatric behavioral health patients in acute medical settings. Child Adolesc Psychiatr Clin N Am.

[18] Luck L, Jackson D, Usher K (2007). STAMP: components of observable behaviour that indicate potential for patient violence in emergency departments. J Adv Nurs.

[19] Gerson R, Malas N, Feuer V, Silver GH, Prasad R, Mroczkowski MM (2019). Best practices for evaluation and treatment of agitated children and adolescents (BETA) in the emergency department: consensus statement of the American Association for Emergency Psychiatry. West J Emerg Med.

[20] Dalton EM, Worsley D, Krass P (2023). Factors influencing agitation, de-escalation, and physical restraint at a children’s hospital. J Hosp Med.

[21] (2019). Critical crossroads: pediatric mental health care in the emergency department. Health Resources & Services Administration.

[22] Shah H, Somaiya M, Chauhan N, Gautam A (2023). Clinical practice guidelines for assessment and management of children and adolescents presenting with psychiatric emergencies. Indian J Psychiatry.

[23] Roppolo LP, Morris DW, Khan F (2020). Improving the management of acutely agitated patients in the emergency department through implementation of Project BETA (Best Practices in the Evaluation and Treatment of Agitation). J Am Coll Emerg Physicians Open.

[24] Latif F, Patel S, Badolato G (2020). Improving youth suicide risk screening and assessment in a pediatric hospital setting by using The Joint Commission guidelines. Hosp Pediatr.

[25] Scudder A, Rosin R, Baltich Nelson B, Boudreaux ED, Larkin C (2022). Suicide screening tools for pediatric emergency department patients: a systematic review. Front Psychiatry.

[26] Lowry NJ, Goger P, Hands Ruz M, Ye F, Cha CB (2024). Suicide risk screening tools for pediatric patients: a systematic review of test accuracy. Pediatrics.

[27] The Joint Commission National Patient Safety Goal on Suicide Prevention in Health Care Settings.

[28] Gvion Y, Apter A (2011). Aggression, impulsivity, and suicide behavior: a review of the literature. Arch Suicide Res.

[29] von Elm E, Altman DG, Egger M (2007). The Strengthening the Reporting of Observational Studies in Epidemiology (STROBE) statement: guidelines for reporting observational studies. Bull World Health Organ.

[30] Collins GS, Moons KG, Dhiman P (2024). TRIPOD+AI statement: updated guidance for reporting clinical prediction models that use regression or machine learning methods. BMJ.

[31] O’Brien BC, Harris IB, Beckman TJ, Reed DA, Cook DA (2014). Standards for reporting qualitative research: a synthesis of recommendations. Acad Med.

[32] Macaluso M, Summerville LA, Tabangin ME, Daraiseh NM (2018). Enhancing the detection of injuries and near-misses among patient care staff in a large pediatric hospital. Scand J Work Environ Health.

[33] Vaughn LM, Lohmueller M (2014). Calling all stakeholders: group-level assessment (GLA)-a qualitative and participatory method for large groups. Eval Rev.

[34] Vaughn LM (2025). Group level assessment methodology as a liberating structure within qualitative and participatory research. Qual Health Res.

[35] Vaughn LM, DeJonckheere M (2019). Methodological progress note: group level assessment. J Hosp Med.

[36] Moser A, Korstjens I (2022). Series: practical guidance to qualitative research. Part 5: co-creative qualitative approaches for emerging themes in primary care research: experience-based co-design, user-centred design and community-based participatory research. Eur J Gen Pract.

[37] Smith S, Winkler S, Towne S, Lutz B (2020). Utilizing CBPR Charrette in community-academic research partnerships – what stakeholders should know. J Particip Res Methods.

[38] Sutton SE, Kemp SP (2006). Integrating social science and design inquiry through interdisciplinary design charrettes: an approach to participatory community problem solving. Am J Community Psychol.

[39] Jones J, Hunter D (1995). Consensus methods for medical and health services research. BMJ.

[40] Cantrill JA, Sibbald B, Buetow S (1996). The Delphi and nominal group techniques in health services research. Int J Pharm Pract.

[41] Haldane V, Koh JJ, Srivastava A (2019). User preferences and persona design for an mHealth intervention to support adherence to cardiovascular disease medication in Singapore: a multi-method study. JMIR Mhealth Uhealth.

[42] Husain L, Finlay T, Husain A, Wherton J, Hughes G, Greenhalgh T (2024). Developing user personas to capture intersecting dimensions of disadvantage in older patients who are marginalised: a qualitative study. Br J Gen Pract.

[43] LeRouge C, Ma J, Sneha S, Tolle K (2013). User profiles and personas in the design and development of consumer health technologies. Int J Med Inform.

[44] Vaughn LM, DeJonckheere M, Pratap JN (2017). Putting a face and context on pediatric surgery cancelations: the development of parent personas to guide equitable surgical care. J Child Health Care.

[45] Braun V, Clarke V (2022). Thematic analysis: a practical guide. QMiP Bull.

[46] Braun V, Clarke V (2006). Using thematic analysis in psychology. Qual Res Psychol.

[47] Cincinnati Children’s Hospital Medical Center Policy Tech CCRF research compliance and oversight record retention and storage. https://cchmc.navexone.com/content/docview/?docid=13534&app=pt&source=unspecified.

[48] Cincinnati Children’s Hospital Medical Center Policy Tech Uses and disclosures of protected health information in research. https://cchmc.navexone.com/content/docview/?docid=13475&app=pt&source=unspecified.

[49] Mesbah H, Rafique Z, Moukaddam N, Peacock WF (2024). Predicting aggressive behavior in psychiatric patients in emergency department: a systematic literature review. Am J Emerg Med.

[50] Anderson KK, Jenson CE (2019). Violence risk-assessment screening tools for acute care mental health settings: literature review. Arch Psychiatr Nurs.

[51] Sammut D, Hallett N, Lees-Deutsch L, Dickens GL (2023). A systematic review of violence risk assessment tools currently used in emergency care settings. J Emerg Nurs.

[52] Kamarova S, Davidson SR, Williams CM, Leite M, Kamper SJ (2025). Predictive validity of violence screening tools in emergency and psychiatric services: a systematic review. Trauma Violence Abuse.

[53] Koh LL, Day A, Klettke B, Daffern M, Chu CM (2020). The predictive validity of youth violence risk assessment tools: a systematic review. Psychol Crime Law.

[54] Mancl ME (2020). Improving Safety in the Pediatric Emergency Department Through Early Violence/Aggression Assessment [doctoral thesis]. https://archive.hshsl.umaryland.edu/server/api/core/bitstreams/2dd81c1a-886d-46a0-9152-285160346cac/content.

[55] Sund G, Kirkpatrick T, Choi KR (2025). Applying the DASA-YV for aggression risk reduction in pediatric acute care. J Pediatr Nurs.

[56] Partridge B, Affleck J (2018). Predicting aggressive patient behaviour in a hospital emergency department: an empirical study of security officers using the Brøset Violence Checklist. Australas Emerg Care.

[57] Mitra B, Settle K, Koolstra C, Talarico C, Smit DV, Cameron PA (2025). Introduction of the Broset Violence Checklist in the emergency department: a retrospective cohort study. Emerg Med Australas.

[58] Huang CJ, Boulos AK, Field S, Wang VJ, Yen K (2024). Workplace violence in the pediatric emergency department: a national survey of physicians in the United States. Pediatr Emerg Care.

[59] Vaughn A, Daraiseh NM, Aeschbury M (2025). Screening tool for predicting patient aggressive behavior and staff injury at a pediatric hospital. J Psychiatr Res.

[60] Martorana JA, Griffith DM, Eiger C (2025). Reducing employee injuries from aggressive patient behavior at children’s hospital by implementing a behavioral response team. Pediatr Qual Saf.

[61] Saidinejad M, Foster AA, Santillanes G (2024). Strategies for optimal management of pediatric acute agitation in emergency settings. J Am Coll Emerg Physicians Open.

[62] Georgadarellis AG, Baum CR (2023). De-escalation techniques for the agitated pediatric patient. Pediatr Emerg Care.

[63] Hoffmann JA, Kshetrapal A, Pergjika A, Foster AA, Wnorowska JH, Johnson JK (2024). A qualitative assessment of barriers and proposed interventions to improve acute agitation management for children with mental and behavioral health conditions in the emergency department. J Acad Consult Liaison Psychiatry.

[64] Hoffmann JA, Johnson JK, Pergjika A, Alpern ER, Corboy JB (2022). Development of quality measures for pediatric agitation management in the emergency department. J Healthc Qual.

