# Supplementary material for: Pediatric Emergency Agitation Care Enhancement: Protocol for a Prospective Mixed Methods Study
Source: JMIR Res Protoc. 2026 Apr 21;15:e92452. doi: 10.2196/92452 (PMC13098726; doi:10.2196/92452)
Supplement: Peer Review Report 1 [file resprot-v15-e92452-s001.pdf]

June 5, 2024

Dear Dr. Babcock,

We are pleased to offer you a Place Outcomes Research Award for your project**,** **"Pediatric Emergency Agitation Care Enhancement (PEACE)**". Your study was innovative and well aligned with the goals of the program. Attached are the reviews from the **(4)** peer reviewers. The review committee had a number of informative comments that may be worth you considering in this project and in your future work.

## Place Outcomes Research Award Summary

**Awardee**: Lynn Babcock
**Project Title**: Pediatric Emergency Agitation Care Enhancement (PEACE)
**Begin Date: July 1, 2024**
**End Date: June 30, 2026**
**Year 1 Award Amount:** $ 100,000

**Year 2 Award Amount:** $ 100,000

***Funding for year two is contingent on satisfactory progress in year one.**

Several important funding issues should be noted. You must have IRB approval before funding can be released. Funds for the second year (if applicable) of the project are contingent on satisfactory progress in year one, as documented by annual progress reports (end of year 1). Finally, please notify us if you are awarded overlapping funds by an outside agency for your project. We may be able to use our internal funds to support other investigators who submitted meritorious awards.

**Sponsored Program Internal Grant Instructions**
Once you have confirmed the start date of your award, please create a Funding Proposal in ePAS Grants. For Place Outcomes Research Grants, you are only required to complete the general information and appropriate ancillary sections (Human Subjects, Animals, Biohazards and Radiation Safety); and the Required Approval sections, excluding the Internal Reviewer. Complete the ePAS Budget Grid and attach the award notification. Once you have obtained the required approvals for the sections listed above, forward to Sponsored Programs Office (SPO Review activity). Sponsored Programs Accounting will then send you an Award Synopsis which will provide you with your new Fund and Project ID (Account Number). If you have any questions regarding this information, please contact your division's SPP representative.

Note: Percent of effort for faculty may be requested up to 10% per year, but at least 1% effort should be recorded for the PI and all Key Personnel in the ePAS budget grid.

Provide an annual progress report as articulated in a forthcoming communication that will contain necessary elements needed for reporting purposes.

If you have any questions regarding this information, please contact your division's SPO representative.

Congratulations once again. Please do not hesitate to contact Heather Kaplan and Pat Brady for any questions or clarification.

Sincerely,

**Heather Kaplan, MD, M.S.C.E**
Attending Neonatologist
Professor, Department of Pediatrics
James M. Anderson Center for Health Systems Excellence
Division of Neonatology and Pulmonary Biology
Cincinnati Children's Hospital Medical Center
3333 Burnet Ave | ML 7009 | Cincinnati, OH 45229
Phone: 513-803-0478 | Fax: 513-803-0969
Heather.Kaplan@cchmc.org

**Patrick W. Brady, MD, MSc**
Interim Co-Director & Research Director, Division of Hospital Medicine
Professor, Department of Pediatrics
Division of Hospital Medicine and
James M. Anderson Center for Health Systems Excellence
Cincinnati Children's Hospital Medical Center
3333 Burnet Ave | ML 9016 | Cincinnati, OH 45229
Phone: 513-636-3635 | Fax: 513-803-9244
Patrick.Brady@cchmc.org

## Place Outcomes Research Award

**Review/Score Sheet**

**Applicant: Lynn Babcock, MD**

Project Title: Pediatric Emergency Agitation Care Enhancement (PEACE)

## Score Guide

| Score | Descriptor | Additional Guidance on Strengths/Weaknesses |
|---|---|---|
| 1 | Exceptional | Exceptionally strong with essentially no weaknesses |
| 2 | Outstanding | Extremely strong with negligible weaknesses |
| 3 | Excellent | Very strong with only some minor weaknesses |
| 4 | Very Good | Strong but with numerous minor weaknesses |
| 5 | Good | Strong but with at least one moderate weakness |
| 6 | Satisfactory | Some strengths but also some moderate weaknesses |
| 7 | Fair | Some strengths but with at least one major weakness |
| 8 | Marginal | A few strengths and a few major weaknesses |
| 9 | Poor | Very few strengths and numerous major weaknesses |

**No decimals please**

**Overall Impact**

Reviewer 1 (2): This is an innovative grant proposal submitted from established investigators to examine full a prediction tool for ARI in children and adolescents presenting to the ED. The investigators previously demonstrated that the tool had good performance. The successful completion of this study has potential to create new knowledge and can be used to predict patient aggression in ED. Aim 1 will test the shortened prediction tool in a prospective cohort study and aim 2 will utilize qualitative methods to create a management plan.  Aim 2 is well described and the investigators have the expertise to carry this out. Some limitations to the proposed study include, aim 1: lack of details surrounding blocks for identifying the study population. More information surrounding outcome variable including how ED staff manage patients who previously needed to be restrained. Also details around time under restraint could be a secondary outcome worth exploring. Additionally, it was difficult to understand the many acronyms used in this application. I was confused between the use of the BVC measure.

Reviewer 2 (2): Mental and behavioral health concerns are among the most common reasons for emergency department visits in children, and the risk to patient and staff safety is significant. Validated tools to identify pediatric patients at risk for agitation and escalation to harm are not available for the ER setting. The proposal concerns priority areas related to clinical and quality of life outcomes and safety. The proposal aims to evaluate an approach to stratify patients based on risk factors to promote early identification and management of agitation, thereby enhancing quality of care, and improving safety for patients and providers. The research team is highly qualified and has collaborated; the Co-Is have developed the brief BRACHA tool and staff safety questionnaire, and the research plan is highly feasible and thoroughly designed using a mixed-methods approach.

Reviewer 3 (3): The study fills a significant gap in the literature by seeking to evaluate the ability of the BRACHA-S tool to assess agitation in mental and behavioral health patients in the ED and create a risk-based management plan to deliver appropriate intervention based on patient agitation and aggression. The team is extremely strong in regard to their expertise and many have previously collaborated together on projects. While the background and innovation sections are extremely strong, there are some key details lacking in the approach that make it difficult to assess how feasible the project truly is. This decreases excitement for the project slightly, but the team also seems strong and would likely be able to navigate potential issues.

Reviewer 4 (3): In brief, Dr. Babcock and colleagues seek to fill a gap in risk stratification and prediction for children presenting to emergency departments (ED) with mental and behavioral health (MBH) challenges. Specifically, they seek to more efficiently, and expeditiously, identify patient populations at risk for agitation that could result in unsafe conditions for patients, families, and staff. They expect that such a tool could be used to interview earlier in the emergency course, potentially reducing the need for medical and physical restraints and potentially reducing unsafe outcomes and injuries. They propose two specific aims, one quantitative and one qualitative: 1) to evaluate the predictive ability of BRACH-S administered by non-MBH specialists early in the ED course for assessing agitation/aggression in children with MBH concerns by conducting a prospective cohort study; and 2) to develop a multi-faceted tiered risk-based management plan designed to mitigate agitation/insight. Aim 2 will involve several qualitative methods, including group level assessment, interviews, and design workshops. This proposal is significant in that it addresses a growing problem in emergency care. It also seeks to use the least restrictive control of aggressive behavior, an important factor in promoting trauma-informed care. Given data suggesting inequities in restraint use, there is also the possibility that the work will address equity gaps. The team assembled is well suited to and well positioned for completing the proposed aims. Strong team with diverse expertise, track record of impact. Important problem with opportunity to make generalizable impact. Mixture of methods, with inclusion of both quantitative and qualitative insights sought. Qualitative aims seeks to include diverse views in creation of risk management plan. I found aspects of the proposal confusing, including the intersection of the BRACHA-S and the BVC. It was not fully clear as to how the methods would be truly mixed. May consider addition of other team members (e.g., BMCP, BST, DDBP, Child Life).

1. **Importance**

Reviewer 1 (1): The benefits of such a study are vast and such enhance the care of children with MBH concerns and the potential for aggressive behavior by recognizing

those at an elevate risk. Such insights with shortened BRACHA could pave the way for predicting children here agitation needs intervention and then create a risk-based management plan that is cocreated with caregivers and ED staff. The authors have preliminary data BRACHA-S has good performance in limited setting.

Reviewer 2 (2): Focus area of high significance and lacking current approaches for identification and management in the pediatric setting.  The tool proposed is not particularly innovative, but has been researched retrospectively and needs prospective validation.  If successful, would be feasible to implement.

Reviewer 3 (2): The argument for why a tool is needed to assess agitation risk in the 3500 children that present with mental and behavioral health concerns is clear and compelling because this could have significant impact on patient outcomes, safety, and resource allocation. There are currently no triage tools for identifying patients who may be aggressive towards themselves, others, or property in the ED – so the current study would fill that gap. Critical pilot work has been conducted to identify a shortened version of the BRACHA that could be feasibly implemented in the ED with a potentially large impact. The possible components of a tiered risk care plan are included (e.g., de-escalation, medication) and similar plans have been implemented in other patient populations to ensure optimal treatment and increase patient outcomes. This care plan would also address how/when to appropriately use physical/mechanical restraints in all patients. The project is innovative by seeking to shift triage of MBH patients by utilizing feasible assessment tools and then creating a care plan based on stakeholder feedback that is actively seeking to provide care with the least restrictive means. Would be helpful to have more information on the pilot study done on the BRACHA-S especially the nature of the ARI incidents in the large sample, which may impact the care plan that is ultimately developed in the current study.

Reviewer 4 (2): Aggression and agitation in ED settings is a significant problem. The investigators do a nice job enumerating the breadth of this problem, the commonality of MBH concerns in Eds and the risk of agitation for the safety of patients, families, and staff. They also effectively acknowledge the complexities surrounding ED care for patients with acute MBG concerns, including the challenges monitoring and addressing escalating behavior. The length of stay within ED settings can lead to escalation that requires steadily increasing invasiveness of responses (verbal to pharmacologic to physical). Clearly, such agitation poses undue risks to all involved in the care of patients. The investigators go on to detail the gap that they hope to fill. They introduce the BRACHA screening tool, a 14-item tool they suggest is too long to be used efficiently. It is not clear to me how much time will be saved in going from the BRACHA to the BRACHA-S (5 items). The BRACHA-S does seem shorter but the time savings may not be significant. That said, if it is efficiently used in an acute setting where every minute count, such time savings could be significant. The link to existing instruments (e.g., BVC) could similarly enhance utility and likelihood of ultimate design of risk-based care algorithms.

The investigators also highlight certain innovative aspects of their proposed project. Specifically, they detail the prospective testing of the shortened BRACHA-S in real-world ED triage settings. They highlight how they will look to use the tool earlier in ED care. Methods that leverage insights from multiple system members add to my enthusiasm. It seems the use of such methods for this purpose is innovative. Significant problem with

safety, equity, quality, and more implications. Strong preliminary data increases feasibility. Stated gap, lack of validated triage tools could be filled by their effort. Potential impact on decreasing aggressive restraints, potentially narrowing equity gaps. Unclear how much time will be saved in going from a 14-item screen to a 5-item screen. Use of the BVC still somewhat unclear (seems to me that it is used as an outcome as an earlier indicator of violent behavior, ideally before violent behavior comes to pass). Certain methods stated aren't particularly innovative in and of themselves though applied to this problem may be innovative.

2. **Rigor**

Reviewer 1 (3):The investigators use varied types of methods including prospective collection of BRACHA-S prediction and the qualitative aim where management plan is co-produced with patients and stakeholders. The qualitative aim is very well described.The investigators use the primary outcome of ARI which is either requires pharmacologic management or physical restraint to main safety. I wonder how physical restraint is chosen over pharmacologic and if the authors should categorize these.Patient recruitment with random block is not well described, especially how it relates to CRC coverage. The BRACHA-S time after patient arrives in ED is vague and described as "shortly after". The authors claim that BRACHA is currently not administered earlier enough after patients arrive, how will the investigators determine what early enough is in this study, they have not defined it. Will the investigators take into account if a patient has a previous ARI, and how does that factor into the prediction?

Reviewer 2 (2): The design is mixed methods, with clear quantitative measures outlined in Aim 1 to validate the brief tool for use in the ED. Aim 2 uses qualitative, participatory action methods to develop management plans aligned with family goals and needs. The plan for recruitment, numbers needed based on power analysis using preliminary data, and statistical plans are well articulated and appropriate. Alternative approaches were outlined if enrollment or staff uptake are insufficient. No significant concerns.

Reviewer 3 (5): The approach section is clearly written and all major components are included. While the necessary details are included in the Study Conduct section for Aim 1, it is difficult to get an understanding of how the study would flow based on the personnel on the study and the unpredictable nature of being able to acquire data on patients in the ED and the staff who are working with them. Data analysis plan for Aim 1 and Aim 2 are strong. Table 2 clearly outlines the approach for Aim 2. The statement of impact outlines how the current proposal fits with the aims of PORA funding including its focus on mental health, clinical and quality of life outcomes, and safety. The timeline is helpful, but it would be helpful to understand how many patients can be recruited each week or month. Would be helpful to additional details/clarification on issues including: What is the rationale for doing repeat BVC scores every 3-4 hours? Are all interventions that are administered in the ED tracked (e.g., verbal de-escalation)? While mentioned in the potential issues and solutions section, data collection is highly dependent on CRCs and it seems like it may be logistically difficult for them to collect the BRACHA-S, BVC, and injury data from staff. It

would be helpful to have a figure or table that shows the flow of the study from patient presenting at the ED, assessment of BRACHA-S and BVC, tracking of interventions, etc. until the patient is discharged. What is the feasibility of recruiting 472 patients across 1 year with the only a 45% effort ED Research Coordinator? Are you planning on enrolling patients across the four score groups? How will this be done to ensure sample size estimates are met? For Aim 2 – will patients be recruited from those whose BRACHA-S and BVC were tracked? How will it be ensured that those who are recruited have lived experiences or expertise in this area? For the PDW sessions, will clinicians also be included as stakeholders? Similarly, how will participants for the 10 key informant interviews be chosen? What will the staff training on the BRACHA-S and BVC entail and how will this be assessed over time? The Nurse Champion will clearly play a critical role, but there is little information about this in the proposal and they only have 5% dedicated time. Are there funding mechanisms for the next step in this research? Or is the next step to implement it into the routine care in the ED at CCHMC?

Reviewer 4 (4): The applicants detail two aims, one quantitative and one qualitative. The first aim purports to use a prospective cohort design, conducted in the real-world ED setting. The study will involve the deployment of the BRACH-S early in the ED course via nurse triage. Patients will then be followed for their ED course. The primary outcome will be agitation/aggression requiring intervention (ARI) as defined by pharmacologics, agitation orderset, and restraint orders. Each of these measures will be captured in Epic. They will also measure staff injuries via reports generated from questionnaires distributed to staff involved in a patient's care. They will be surveyed once per shift (though not clear when in the shift and whether there will be any weighting based on time spent with patient, i.e., is a no from someone who saw a patient once for a few minutes the same as a no from someone who was with a patient for an 8-hour shift?). The team will also explore a meaningful change in the BVC measure, a questionnaire that will be repeated at regular interviews (q3-4 hours). It is not clear if this will be a narrow enough time window to see effect. The analytic plan, however, does seem appropriate, though I wonder a bit whether survival methods (time to first ARI) could be effective. I also wonder about whether a directed acyclic graph could be helpful in defining linkages between potential covariates and key exposures and outcomes.

The second aim uses a variety of qualitative methods to develop a risk-based tiered management algorithm, designed to promote the least restrictive restraint methods. Clear strengths include inclusion of a diverse population and use of rigorous qualitative methods to move toward action plans. The use of personas will likely help stimulate valuable conversations and could facilitate creation of a consensus risk prediction tool and care plan bundle. My enthusiasm would be higher if this potential bundle were made a bit more tangible. Understanding that the design process will ultimately result in the bundle, I still wondered what the applicants envision this could be? How could it be implemented? Breadth of measures available (or planned) to collect during quantitative aim. Assessment of inter-rater reliability between nurses and MBG specialists. Mixture of quantitative and

qualitative methods. Diverse stakeholders included in the qualitative methods. Certain measures (e.g., BVC, staff injury) may not be collected frequently enough to provide precise estimates. Could benefit from a directed acyclic graph, or conceptual model, to elicit when and how to evaluate certain variables. This may be addressed by the inter-rater reliability assessments, but I do wonder if there will be analyses that cluster by or adjust for staff, time of day, busy-ness of the ED. Not fully clear how the methods will be truly mixed. Unclear as to what a potential care bundle could look like. What 'technical supports' might the team implement to overcome logistical challenges? E.g., child care, transportation supports, virtual/digital supports, etc.

3. **Expertise**

Reviewer 1 (2): The investigators bring a wealth of demonstrated expertise in the fields of Emergency Medicine, psychiatry, and qualitative experts, as well as nursing investigators. Their collective experience spans extensive research, clinical practice, and academic contributions in these disciplines. PI prior work is mostly in TBI. I am curious as to why a CHOP investigator is included as a consultant on this grant.

Reviewer 2 (1): The PI and Co-Is are highly qualified, well suited to the topic and study designed, and have collaborated clinically, and on prior research. Plans for staff, including clinical and research coordinators is evident. Letters of support document the commitment of collaborators in the emergency department and for the tools proposed. No concerns.

Reviewer 3 (1): PI has the experience/expertise based on clinical and research background and recently completed CDC study on use of the MOST in adolescents with mild TBI. Dr. Babcock also just recently refined the BRACHA tool to a concise 5-item version that can be used for triage in the ED. Team consists of PI (Babcock), Co-Is with expertise in the BRACHA tool and management of acute agitation (Barzman), qualitative data and collaboration with QMAC (Vaughn), and injury prevention (Pomerantz), and other investigators with expertise in statistics (Zhang), injury prevention, violence assessment (Broset Violence Checklist), and use of restraints (Hanson), nursing (Siders), pulling EMR data (Murphy), assessing and analyzing staff injuries (Dariaseh). Dr. Ketabchi at CHOP previously helped with this work when he was a fellow at CCHMC and will continue to provide expertise. Many of the team members have actively collaborated on previous studies and have experience conducting data with EMR records and the use of the BRACHA tool in the psychiatric hospital setting. Will have a dedicated nurse champion and 2 CRCs. The proposal and budget justification very clearly lay out the roles for each investigator and other key personnel. Collaborating with both DMAC and QMAC for data analysis.

Reviewer 4 (2): Very strong, accomplished team members. Diverse expertise. Committed to project. Some individuals have just a very small FTE (1 or 2%) assigned to project. Nurse champion not yet identified. Could benefit from involvement of BMCP, BST, DDBP, potentially other services/supports.Could benefit from parent partner on core research team.

**Application scores**

- Composite score: 2.5
- Overall score: 2.5
